# Disease-free and overall survival at 3.5 years for neoadjuvant bevacizumab added to docetaxel followed by fluorouracil, epirubicin and cyclophosphamide, for women with HER2 negative early breast cancer: ARTemis Trial

**DOI:** 10.1093/annonc/mdx173

**Published:** 2017-04-27

**Authors:** H. M. Earl, L. Hiller, J. A. Dunn, C. Blenkinsop, L. Grybowicz, A.-L. Vallier, I. Gounaris, J. E. Abraham, L. Hughes-Davies, K. McAdam, S. Chan, R. Ahmad, T. Hickish, D. Rea, C. Caldas, J. M. S. Bartlett, D. A. Cameron, E. Provenzano, J. Thomas, R. L. Hayward, for the ARTemis Investigators Group

**Affiliations:** 1 Department of Oncology, University of Cambridge, Cambridge;; 2 NIHR Cambridge Biomedical Research Centre, Cambridge;; 3 Cambridge Breast Cancer Research Unit, Cambridge University Hospitals NHS Foundation Trust, Cambridge;; 4 Warwick Clinical Trials Unit, University of Warwick, Coventry;; 5 Cambridge Clinical Trials Unit - Cancer Theme, Cambridge University Hospitals NHS Foundation Trust, Cambridge;; 6 Department of Oncology, Queen Elizabeth Hospital King's Lynn NHS Foundation Trust, King's Lynn;; 7 Cancer Research UK Cambridge Institute, University of Cambridge, Cambridge;; 8 Department of Oncology, Cambridge University Hospitals NHS Foundation Trust, Cambridge;; 9 Department of Oncology, Peterborough and Stamford Hospitals NHS Foundation Trust, Peterborough;; 10 Department of Oncology, Nottingham City Hospital, Nottingham;; 11 Department of Oncology, West Middlesex University Hospital, Isleworth;; 12 Department of Oncology, Poole Hospital NHS Foundation Trust/Bournemouth University, Poole;; 13 Cancer Research UK Clinical Trials Unit, University of Birmingham, Edgbaston, UK;; 14 Ontario Institute for Cancer Research, MaRS Centre, Toronto, Canada;; 15 Cancer Research Centre, University of Edinburgh, IGMM, Western General Hospital, Edinburgh;; 16 Department of Histopathology, Cambridge University Hospitals NHS Foundation Trust, Cambridge;; 17 Department of Pathology, University of Edinburgh, Edinburgh, UK

**Keywords:** ARTemis, breast cancer, bevacizumab, neoadjuvant chemotherapy

## Abstract

**Background:**

The ARTemis trial previously reported that addition of neoadjuvant bevacizumab (Bev) to docetaxel (D) followed by fluorouracil, epirubicin and cyclophosphamide (D-FEC) in HER2 negative breast cancer improved the pathological complete response (pCR) rate. We present disease-free survival (DFS) and overall survival (OS) with central pathology review.

**Patients and methods:**

Patients were randomized to 3 cycles of D followed by 3 cycles of FEC (D-FEC), ±4 cycles of Bev (Bev + D-FEC). DFS and OS were analyzed by treatment and by central pathology reviewed pCR and Residual Cancer Burden (RCB) class.

**Results:**

A total of 800 patients were randomized [median follow-up 3.5 years (IQR 3.2–4.4)]. DFS and OS were similar across treatment arms [DFS hazard ratio (HR)=1.18 (95% CI 0.89–1.57), *P *=* *0.25; OS HR = 1.26 (95% CI 0.90–1.76), *P *=* *0.19). Both local pathology report review and central histopathology review confirmed a significant improvement in DFS and OS for patients who achieved a pCR [DFS HR = 0.38 (95% CI 0.23–0.63), *P *<* *0.001; OS HR = 0.43 (95% CI 0.24–0.75), *P = *0.003]. However, significant heterogeneity was observed (*P *=* *0.02); larger improvements in DFS were obtained with a pCR achieved with D-FEC than a pCR achieved with Bev + D-FEC. As RCB class increased, significantly worse DFS and OS was observed (*P* for trend <0.0001), which effect was most marked in the ER negative group.

**Conclusions:**

The addition of short course neoadjuvant Bev to standard chemotherapy did not demonstrate a DFS or OS benefit. Achieving a pCR with D-FEC is associated with improved DFS and OS but not when pCR is achieved with Bev + D-FEC. At the present time therefore, Bev is not recommended in early breast cancer.

**ClinicalTrials.gov number:**

NCT01093235.

## Introduction

The ARTemis trial was designed to test the hypothesis that adding bevacizumab (Bev) [1, 2] to standard neoadjuvant chemotherapy would improve pathological complete response rates (pCR), and longer-term outcomes for HER2 negative early breast cancer. Assessed by a two-reader blinded review of local pathology reports, the addition of four cycles of Bev to D-FEC was found to improve pCR rates (22% for Bev + D-FEC patients, 17% for D-FEC patients, adjusted *P *=* *0.03) [3]. Other neoadjuvant trials (GeparQuinto [4], CALGB 40603 [5] and NSABP-B40 study [6]) also showed an improvement in pCR rates with the addition of Bev to neoadjuvant chemotherapy. However, adjuvant Bev in the BEATRICE study in TNBC patients [7] and in the ECOG 5103 study [8], showed no improvement in invasive disease-free survival (IDFS) or overall survival (OS). Both of these adjuvant trials used a year of Bev in the experimental arm, as did the NSABP-B40. In contrast, shorter courses of Bev were used in the other trials: four cycles at 15 mg/kg every 3 weeks (q3w) in ARTemis; eight cycles at 15 mg/kg q3w in GeparQuinto; and nine cycles at 10 mg/kg q2w in CALGB 40603.

A central pathological review of diagnostic and surgical excision histopathology slides was undertaken (manuscript in press 2017) which included Residual Cancer Burden (RCB) class [9]. Using these analyses, we present here the secondary endpoints of DFS and OS for the ARTemis trial to assess whether the increase in pCR rate results in improved longer-term outcomes.

## Methods

ARTemis is an investigator designed and led, open label randomized, phase III trial approved by the South-East England Multi-Centre Research Ethics Committee and the Research and Development departments at all participating centres. It was granted a Clinical Trials Authorization from the Medicines and Healthcare products Regulatory Agency on 25 February 2009. Trial co-ordination was supported by a Cancer Research UK project grant (CRUK/08/037). An unrestricted educational grant and free Bev was provided by Roche and an unrestricted educational grant by Sanofi.

### Study design

Full details of the design, sample size, eligibility, stratification and treatments have been described elsewhere [3]. Eligibility included women with a histological diagnosis of non-metastatic HER2 negative invasive breast cancer, and a radiological tumor size of >20 mm with or without axillary involvement. All patients provided written informed consent and could commence chemotherapy within one week of randomization. Patients with inflammatory cancer, T4 tumors with direct extension to the chest wall or skin, and ipsilateral supraclavicular lymph node involvement were eligible with any size of primary tumor. The two randomized treatments were: three cycles of docetaxel (100 mg/m^2^ once every 21 days) followed by three cycles of fluorouracil, epirubicin, and cyclophosphamide (500 : 100 : 500 mg/m^2^) once every 21 days (D-FEC), with or without four cycles of Bev (15 mg/kg) (Bev + D-FEC) commencing with the first cycle of docetaxel.

### Patients

Patients were randomly assigned (1 : 1) by telephone to the Warwick Clinical Trials Unit. Using a central computerized minimization procedure, stratification was by age (≤50 : >50), ER status [strongly positive (Allred score 6–8): weakly positive (Allred score 3–5): negative (Allred score ≤2)], total tumor size (≤5 cm : >5 cm), clinical involvement of axillary lymph nodes (yes : no) and disease type (inflammatory and/or locally advanced: neither).

### Central pathology specimen review

Two breast pathologists on the trial management group reviewed, blind to local pathology reports and patient outcomes, all collected histopathology slides for response (pCR and RCB) [9].

### Statistical analysis

OS was calculated from date of randomization to date of death from any cause, or date of censoring if alive. DFS was calculated from date of randomization to date of first relapse (loco-regional or distant, not including DCIS); to date of death in women dying without invasive relapse; or to date of censoring in women alive and disease-free. Survival curves were constructed using Kaplan–Meier methodology and assessed using log-rank tests. Cox-proportional hazards modelling was used to investigate treatment effects, whilst adjusting for stratification variables. Hazard ratios of treatment effects on the risk of relapse and death for each of the stratified subgroups were displayed on HR plots [10]. To assess the association between response to neoadjuvant treatment and DFS and OS, a landmark analysis was undertaken recalculating times from date of surgery. Pathological response rates were assessed across randomized treatment arms using *χ*^2^ tests, with continuity correction where appropriate, and logistic regression to adjust for stratification factors.

We report the protocol-stated pre-planned interim analysis of DFS and OS with at least 120 events (median follow-up 3 years). All analyses were undertaken by Warwick Clinical Trials Unit with SAS statistical software (version 9.3). Protocol violators were analyzed within their randomized groups on an intention-to-treat basis. All reported *P*-values are two-sided. ARTemis is registered with EudraCT (2008-002322-11), ISRCTN (68502941), and ClinicalTrials.gov (NCT01093235).

## Results

### Patient characteristics

**Figure 1.**
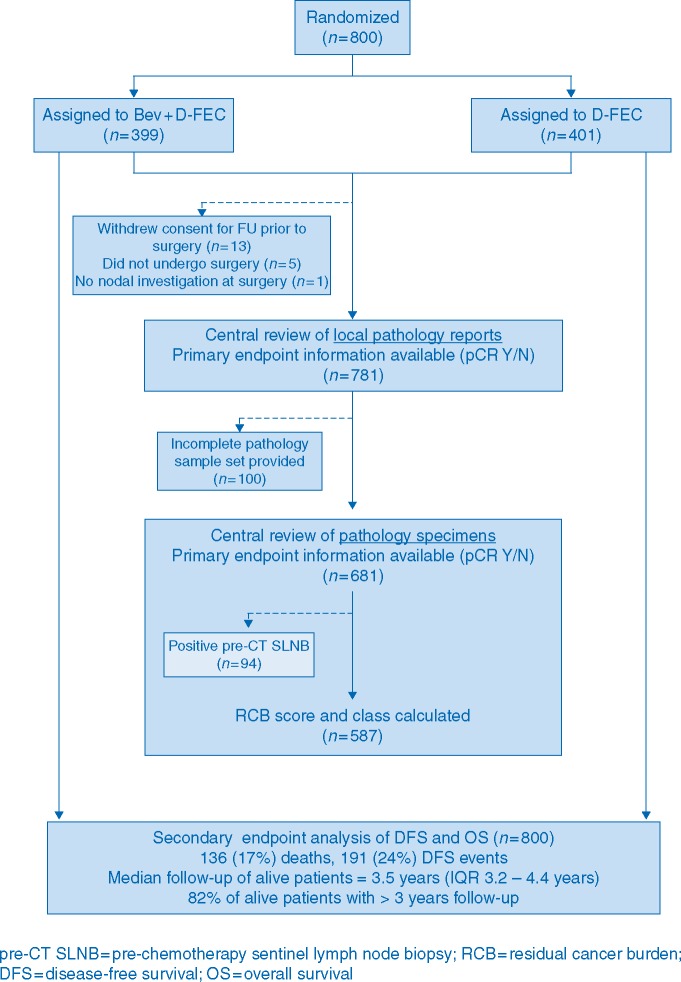
Consort diagram.

A total of 800 patients were randomized into ARTemis between May 2009 and January 2013; 399 to Bev + D-FEC, 401 to D-FEC (Figure 1 and Table 1). Patient characteristics were balanced across randomized treatment arms [3]. The distribution of important prognostic factors in the subgroups with available central pathology review was similar to the full trial (Table 1).

**Table 1:** Patient characteristics and response to treatment

Patient characteristics		Full trial population	Central pathology sample with primary endpoint assessable	Central pathology sample with RCB assessable
		(*n *=800)	(*n *=681)	(*n *=587)
		*n* (%)	*n* (%)	*n* (%)
Randomized treatment	Bev+D-FEC	399 (50)	344 (51)	290 (49)
	D-FEC	401 (50)	337 (49)	297 (51)
Age	≤50 years old	543 (68)	458 (67)	393 (67)
	>50 years old	257 (32)	223 (33)	194 (33)
ER status	Negative (Allred score 0–2)	248 (31)	211 (31)	194 (33)
	Weakly positive (Allred score 3–5)	75 (9)	68 (10)	60 (10)
	Strongly positive (Allred score 6–8)	477 (60)	402 (59)	333 (57)
Tumor size	≤50 mm	635 (79)	541 (79)	472 (80)
	>50 mm	165 (21)	140 (21)	115 (20)
Clinical involvement of	Yes	417 (52)	354 (52)	299 (51)
axillary nodes	No	383 (48)	327 (48)	288 (49)
Inflammatory or locally	Yes	149 (19)	120 (18)	103 (18)
advanced disease or both	No	651 (81)	561 (82)	484 (82)

Response to Treatment				

pCR	Yes	–	130 (19)	121 (21)
	No	–	551 (81)	466 (79)
RCB class	0	–	–	121 (21)
	1	–	–	90 (15)
	2	–	–	290 (49)
	3	–	–	86 (15)

pCR, central pathology sample review shows pathological complete response in all breast tumors AND absence of disease in all removed axillary lymph nodes; RCB, residual cancer burden.

### Central pathology review and pCR rates

The original analysis of the primary endpoint of pCR on the 781 patients who had surgery within the trial used a two-reader independent review of local pathology reports (Figure 1). This allowed detection of absolute differences between treatment arms in the pCR rates >10% at the 5% (two-sided) level of significance (85% power). Histopathology slide retrieval was successful in obtaining a full slide set in 681/781 patients (87%). This ensured that the central pathological review allowed detection of the same 10% differences (power reduced to 80%). Patients with positive pre-treatment sentinel lymph node biopsy (SLNB) were excluded from RCB assessment, as per the guidelines [9] leaving 587/681 patients (86%) with calculated RCB (Figure 1).

In the original publication, based on the 2-reader report review, pCR was reported for 153/781 patients (20%) [3]. For patients who had central pathological review (*n = *681), pCR was reported in 130/681 patients (19%), with a higher pCR rate for Bev + D-FEC patients [77/344 (22%) versus 53/337 (16%) for D-FEC patients; adjusted *P *=* *0.03; Table 2]. Amongst the 587 patients with assessable RCB, treatment with Bev resulted in a shift towards better (lower) RCB classes (adjusted *P* for trend = 0.004; Table 2).

**Table 2:** Response rates from the central review of pathology specimens, across randomized treatment arms

	Bev+D-FEC	D-FEC	
	*n* (%)	*n* (%)	*P* (adjusted *P*^a^)
pCR (*n *=681)			
Yes	77 (22)	53 (16)	0.03 (0.03)
No	267 (78)	284 (84)	
RCB class (*n *=587)			
0	72 (25)	49 (16)	0.004 (0.004)
1	46 (16)	44 (15)	
2	138 (47)	152 (51)	
3	34 (12)	52 (18)	

a Adjusted for the five stratification variables.

pCR, pathological complete response in all breast tumors AND absence of disease in all removed axillary lymph nodes; RCB, residual cancer burden.

### Disease-free and overall survival

At the data lock (14 April 2016), 136/800 (17%) patients had died (Figure 1). The median follow-up for alive patients was 3.5 years, with 82% of alive patients having >3 years follow-up. The main cause of death was breast cancer [98% (133/136) of patients who died]. Seventy-two patients have a local relapse, and 151 patients a distant relapse, predominantly in the bone, liver and/or lung (81% of patients who have a distant relapse). Forty-seven patients reported a local and distant relapse. There are 191 events in the DFS analysis (24%).

**Figure 2.**
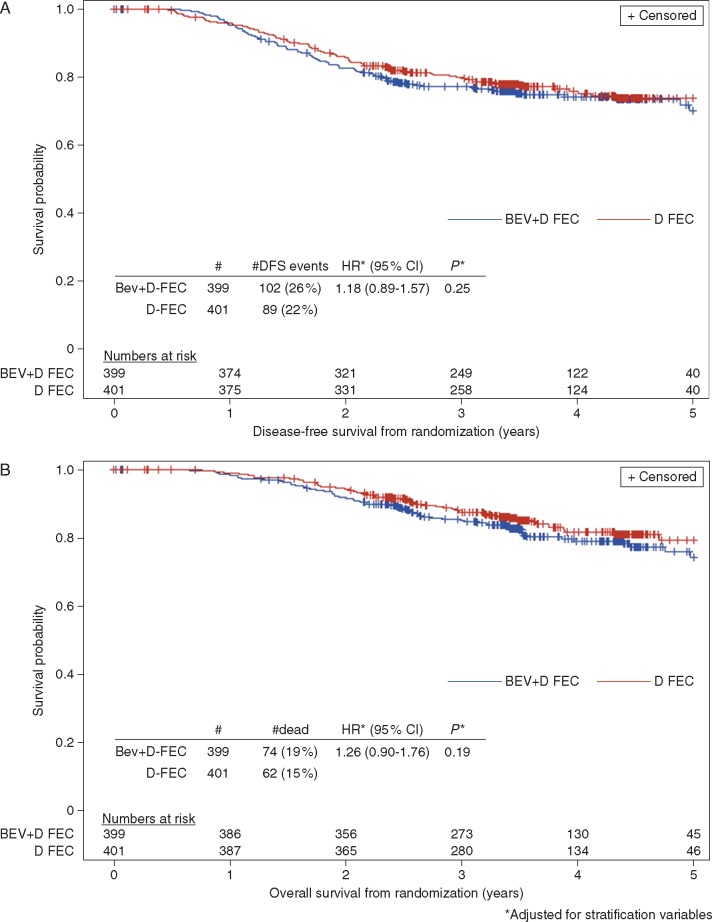
Survival curves by randomized treatment arm. (A) Disease-free survival and (B) Overall survival.

There were no significant differences detected in DFS or OS between the two randomized treatment arms [DFS HR 1.18 (95% CI 0.89–1.57), *P *=* *0.25, Figure 2A; OS HR 1.26 (95% CI 0.90–1.76), *P *=* *0.19, Figure 2B]. There was evidence of heterogeneity only in the treatment effect on DFS for patients with clinically negative nodes at diagnosis (heterogeneity *P *=* *0.02, not adjusted for multiple comparisons) (supplementary Figure S1A, available at *Annals of Oncology* online). Otherwise no heterogeneity was observed in the treatment effect on DFS and OS across all patient characteristics (supplementary Figure S1B, available at *Annals of Oncology* online). However, there appeared to be a slightly worse DFS and OS for ER strongly positive patients treated with Bev (supplementary Figure S2, available at *Annals of Oncology* online).

### DFS and OS from surgery by pCR

The landmark analysis, investigating the effect of pathological response on DFS and OS, included 677/681 patients; 109/677 (16%) subsequently died, and 157/677 (23%) subsequently had a DFS event. Analysis of DFS events in the pCR group (supplementary Table S1, available at *Annals of Oncology* online) demonstrated that, although more patients achieved a pCR in the Bev + D-FEC arm (22% versus 16% for D-FEC), 16/77 (16%) had a DFS event compared with only 3/52 (6%) in the D-FEC arm.

**Figure 3.**
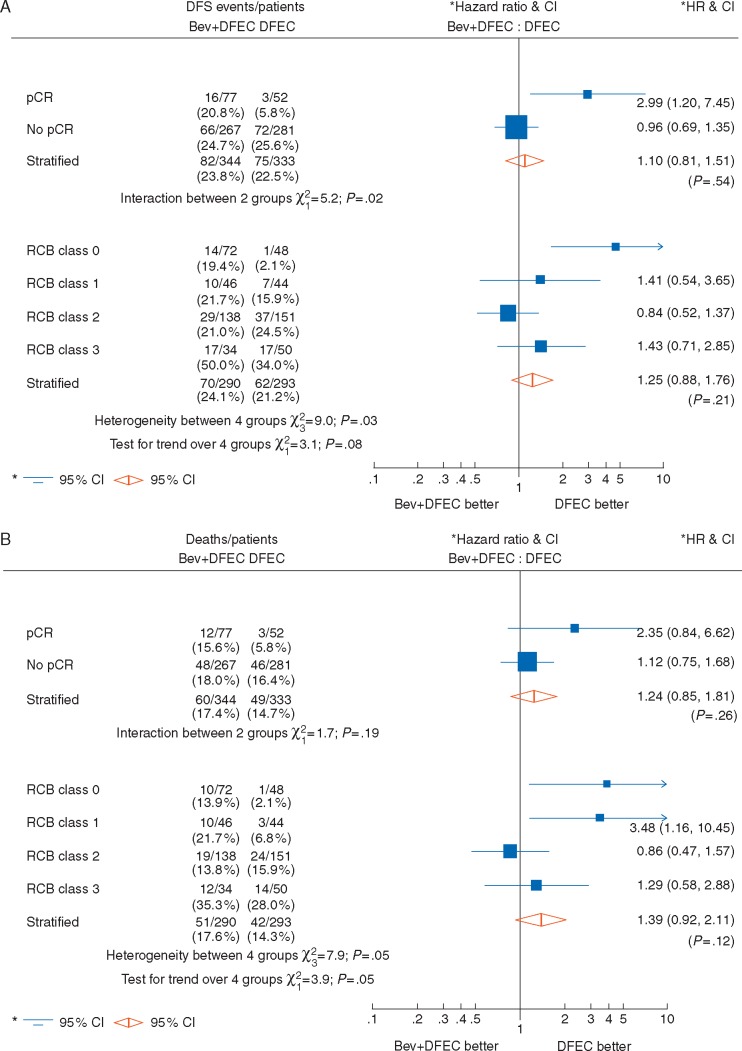
Treatment effect by pathological response. (A) Disease-free survival from surgery and (B) Overall survival from surgery.

There was a significant improvement in both DFS and OS for patients obtaining pCR [DFS HR 0.38 (95% CI 0.23–0.63), *P *<* *0.001; supplementary Figure S3A, available at *Annals of Oncology* online; OS HR 0.43 (95% CI 0.24–0.75), *P *=* *0.003; supplementary Figure S3D, available at *Annals of Oncology* online]. However, there was significant heterogeneity in treatment effect on DFS between patients achieving pCR or not (*P *=* *0.02) and according to RCB class (*P *=* *0.03) (Figure 3A). Importantly, patients achieving pCR in the Bev + D-FEC arm had a risk of a DFS event that was 2.99-fold higher (95% CI 1.20–7.45) than that for patients achieving pCR in the D-FEC arm (Figure 3A). Similar findings, although non-significant, were seen for OS (*P *=* *0.19 for pCR and *P *=* *0.05 for RCB class) (Figure 3B). DFS and OS curves plotted by treatment arm demonstrated this larger improvement in D-FEC patients (supplementary Figure S3C and F, B and E, available at *Annals of Oncology* online). As RCB class increased, significantly worse DFS and OS was observed (both *P* for trend <0.0001, supplementary Figure S4A and D, available at *Annals of Oncology* online) and, similar to pCR, with differing treatment effects across the classes (DFS heterogeneity *P *=* *0.03, Figure 3A; OS heterogeneity *P *=* *0.05, Figure 3B). An additional analysis of DFS and OS by RCB for ER groups is shown (supplementary Figure S5, available at *Annals of Oncology* online).

## Discussion

The ARTemis trial results reported here demonstrate no advantage for short course neoadjuvant Bev in terms of DFS and OS at a median follow-up of 3.5 years and these results are similar to those of GeparQuinto [11] and CALGB-40603 [12]. It has been shown in most neoadjuvant breast cancer trials that longer term outcomes, analyzed by treatment arm, fail to show a benefit even when there are significant improvements in pCR rates. It is now understood that this is due to a complexity of interacting factors [13–15], the most obvious of which is the smaller number of patients required in neoadjuvant trials. Only one neoadjuvant trial in HER2 positive breast cancer adding trastuzumab to standard chemotherapy showed improved long term outcomes by treatment arm [16].

Pooled analyses [17, 18] have shown that patients achieving pCR have significantly better DFS and OS than other patients. However, ARTemis shows that gaining a pCR for patients with the addition of Bev does not appear to have this benefit, and the outcomes for these patients are not significantly better than for those not achieving a pCR. This is clearly demonstrated both by the Kaplan–Meier DFS and OS curves by pCR and treatment arms (supplementary Figure S3, available at *Annals of Oncology* online), and in the forest plots (Figure 3). This result has led to our hypothesis [3] that although Bev improves pCR rates by its effect in the angiogenesis-dependent primary tumor, it has no effect on putative angiogenesis-independent micro-metastatic disease. This hypothesis would also explain the negative long-term results from GeparQuinto and CALGB-40603 [12, 13] and adjuvant BEATRICE and ECOG studies [7, 8]. Similar negative results have been found in adjuvant studies in colorectal cancer [19] and melanoma [20]. In contrast, in epithelial ovarian cancer (EOC) in the first line setting [21, 22] positive long-term results have been shown probably for two reasons; firstly the majority of patients had macroscopic residual disease post-surgery which is angiogenesis-dependent; and secondly there may be an autocrine effect of VEGF directly on receptors on ovarian cancer cells [23].

Intriguingly, the ARTemis data hint at the possibility that patients in the experimental arm do non-significantly but slightly worse than the standard arm (supplementary Figure S1, available at *Annals of Oncology* online). One explanation is the possible increased populations of classically chemo-resistant breast cancer stem cells in tumors due to the hypoxia generated by Bev [24]. In addition, there is possibly a group for whom Bev is having a detrimental effect. This has been reported in EOC where an ‘immunological signature’ with a better prognosis was associated with a negative interaction with Bev [25]. We plan translational research to discover whether there are similar molecular signatures in ARTemis.

Our central pathology review and analysis of RCB classes has provided some interesting additional results. Bev shows a benefit in terms of the proportion of patients achieving pCR, but there is no improvement in survival for patients achieving a pCR. Central review confirms these findings from the two-reader report review [3].

In conclusion the ARTemis trial shows that, although the addition of Bev to taxane-anthracycline-based chemotherapy increases pCR rates, it does not provide a corresponding benefit in terms of DFS and OS.

## Supplementary Material

Supplementary Figure 1a

Supplementary Figure 1b

Supplementary Figure 2abc

Supplementary Figure 2def

Supplementary Figure 3abc

Supplementary Figure 3def

Supplementary Figure 4abc

Supplementary Figure 4def

Supplementary Figure 5abc

Supplementary Figure 5def

Supplementary Table S1
