# Supplementary figures and images for: Disease-free and overall survival at 3.5 years for neoadjuvant bevacizumab added to docetaxel followed by fluorouracil, epirubicin and cyclophosphamide, for women with HER2 negative early breast cancer: ARTemis Trial
Source: Ann Oncol. 2017 Apr 27;28(8):1817–24. doi: 10.1093/annonc/mdx173 (PMC5834079; doi:10.1093/annonc/mdx173)

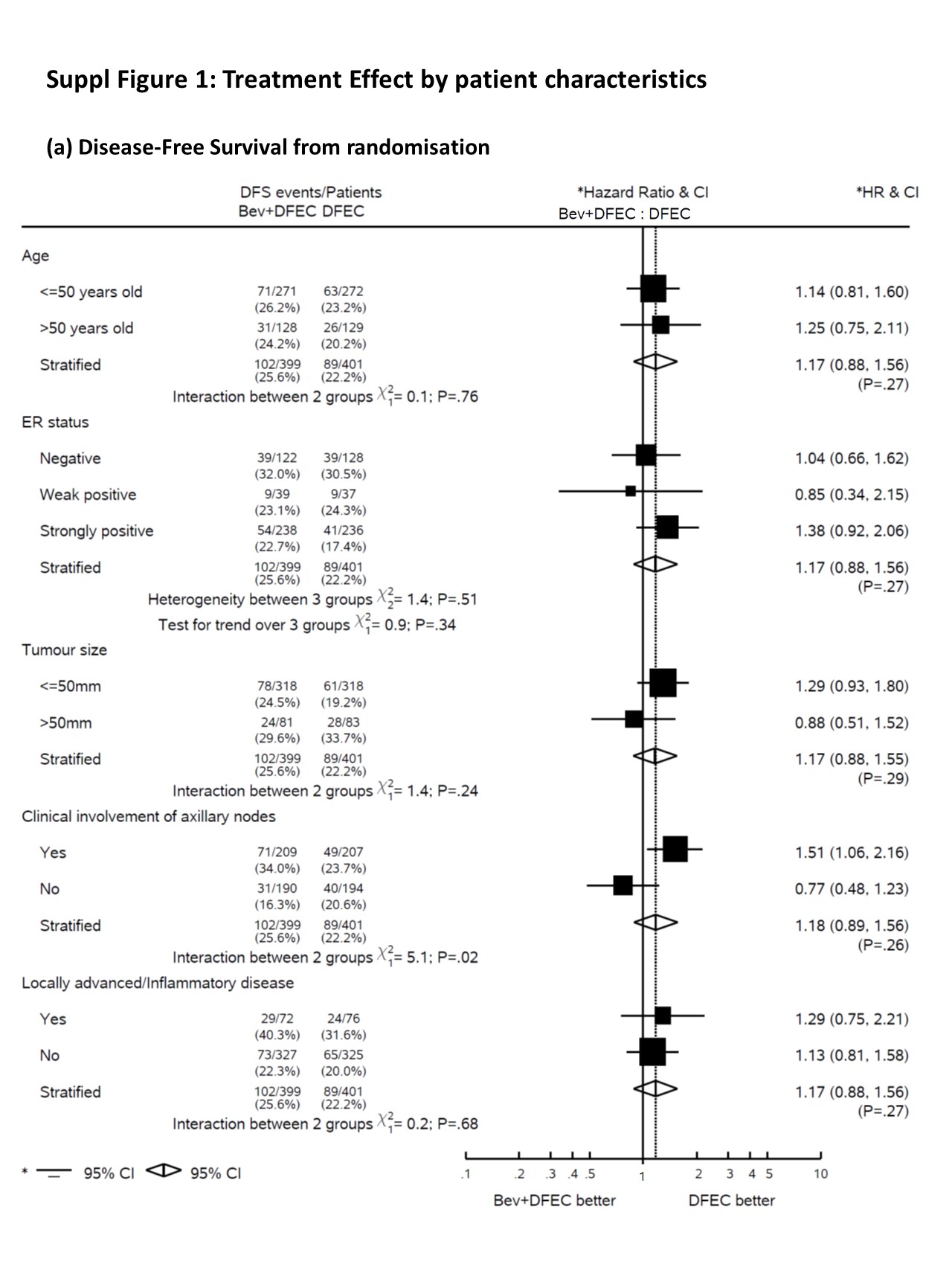

Supplement: Supplementary Figure 1a [file fig_s1a_mdx173.jpeg]

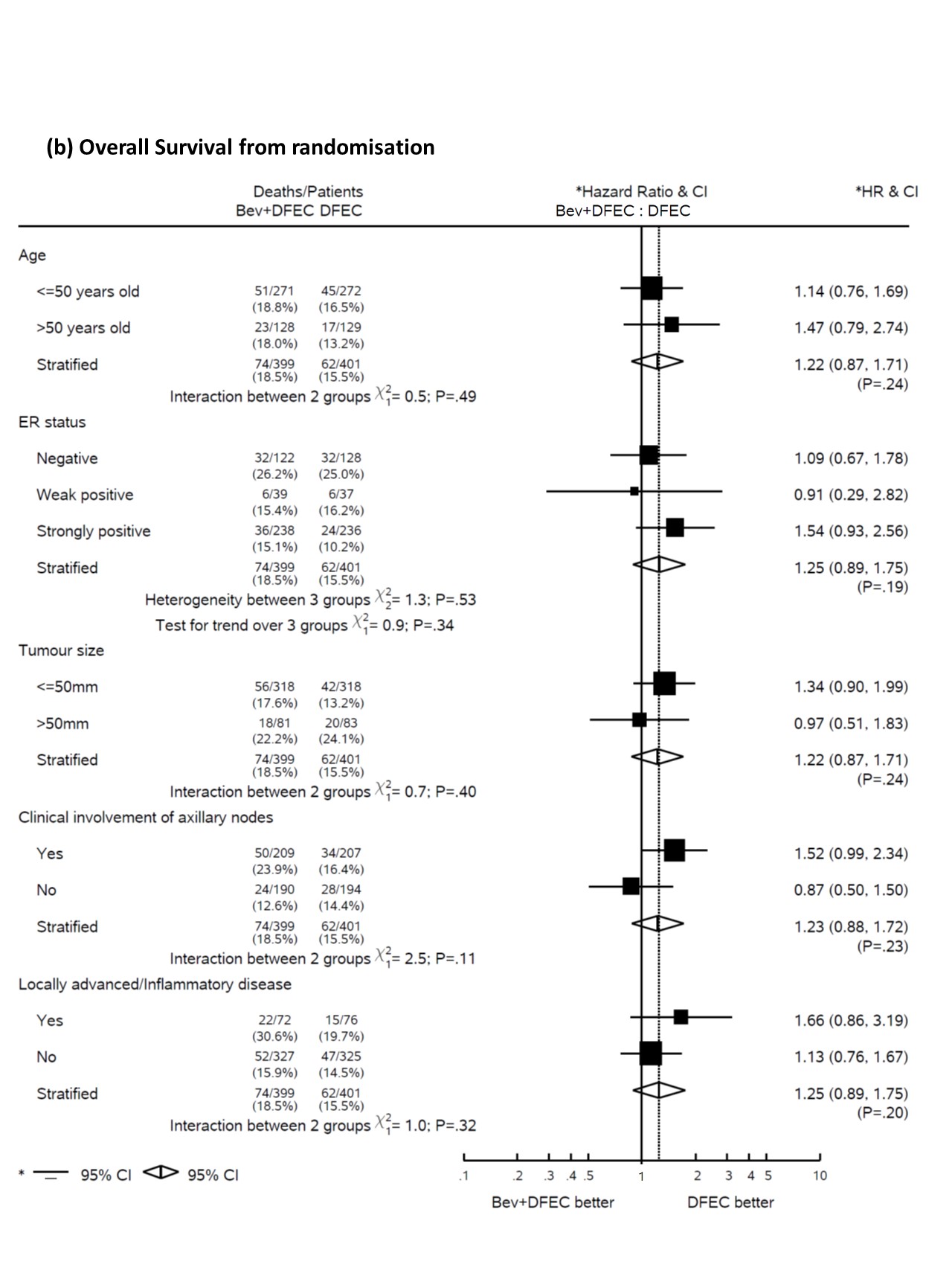

Supplement: Supplementary Figure 1b [file fig_s1b_mdx173.jpeg]

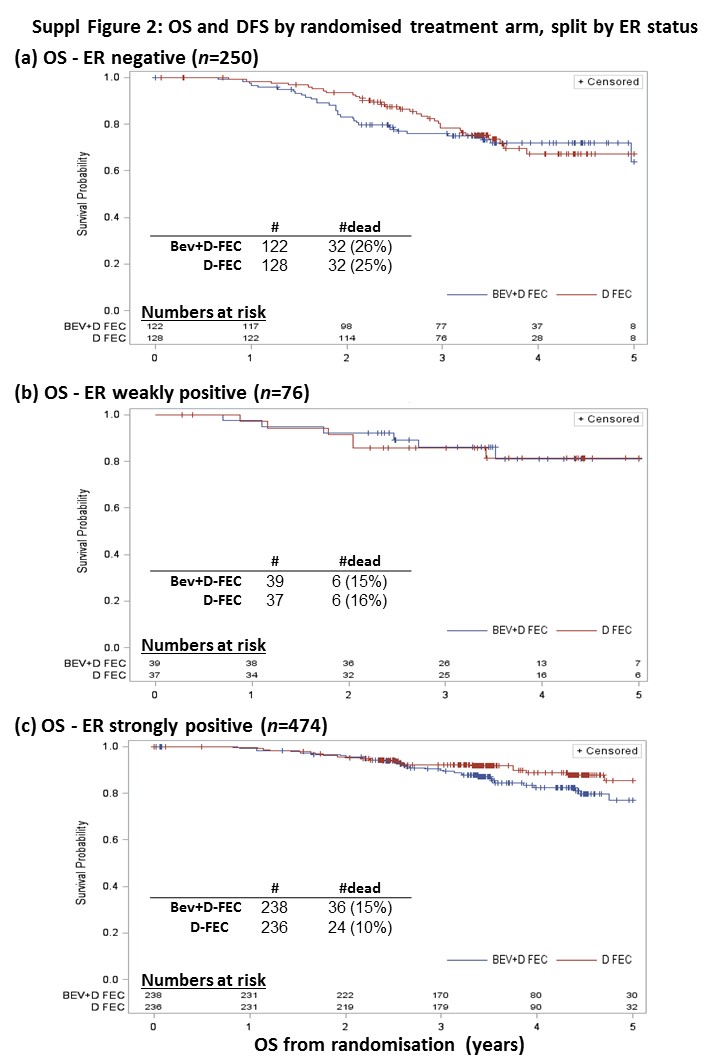

Supplement: Supplementary Figure 2abc [file fig_s2abc_mdx173.jpeg]

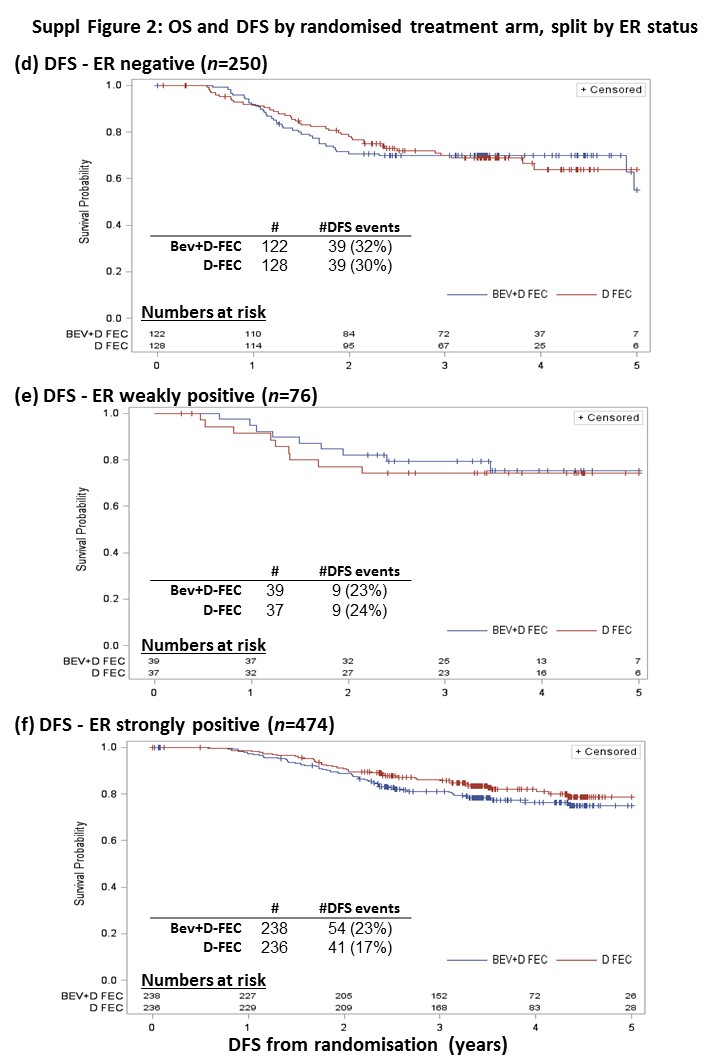

Supplement: Supplementary Figure 2def [file fig_s2def_mdx173.jpeg]

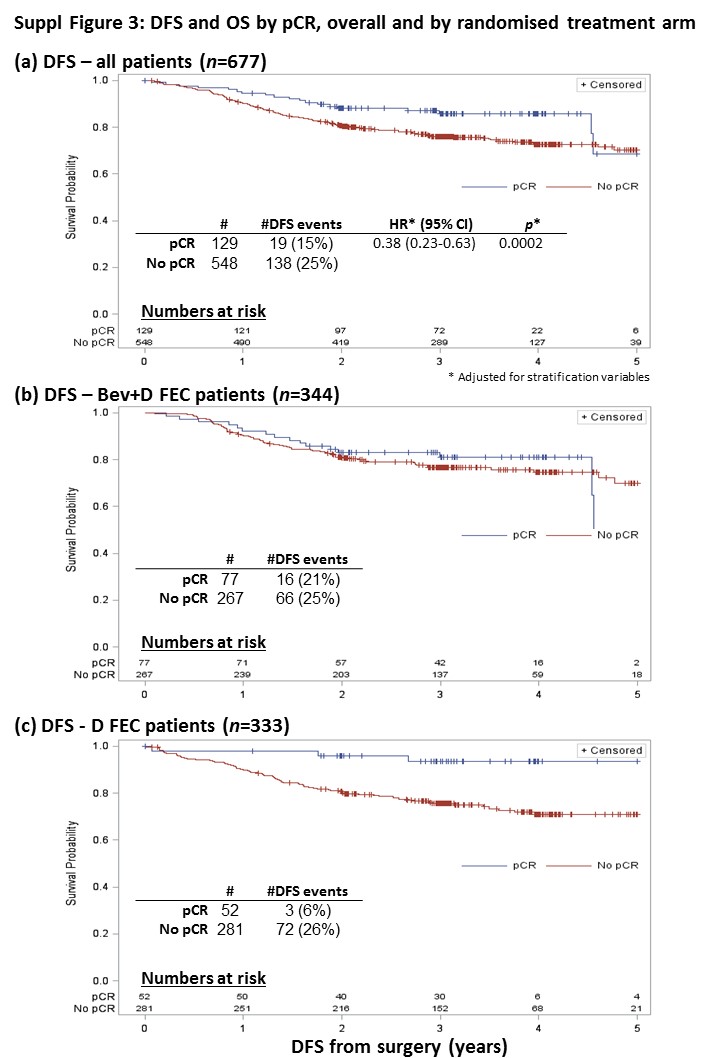

Supplement: Supplementary Figure 3abc [file fig_s3abc_mdx173.jpeg]

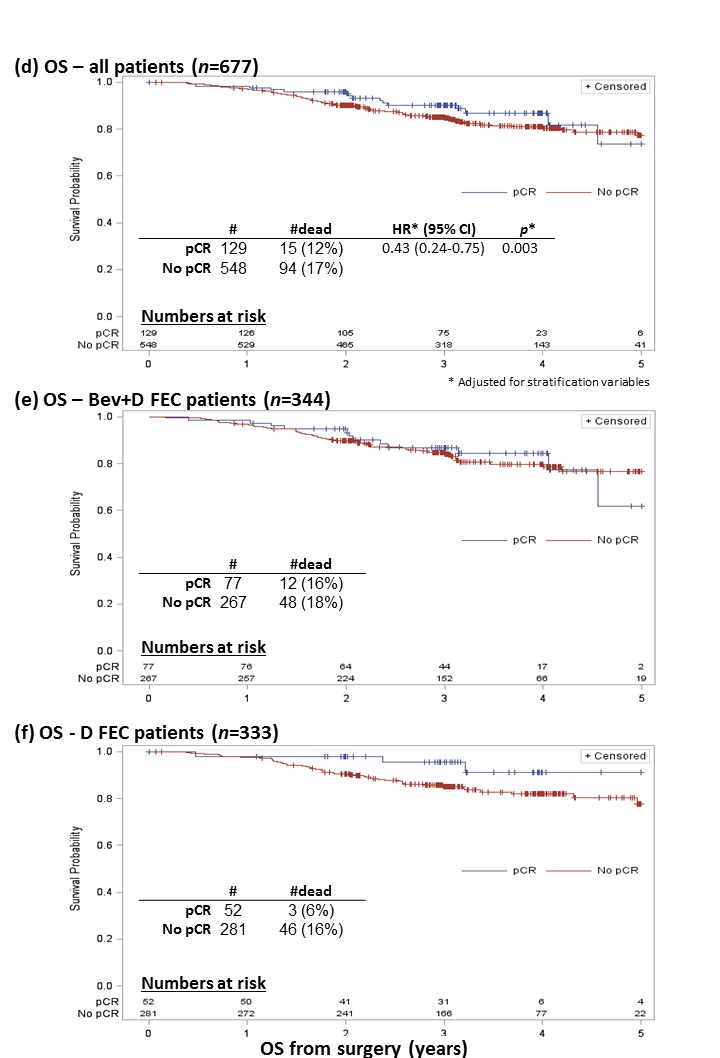

Supplement: Supplementary Figure 3def [file fig_s3def_mdx173.jpeg]

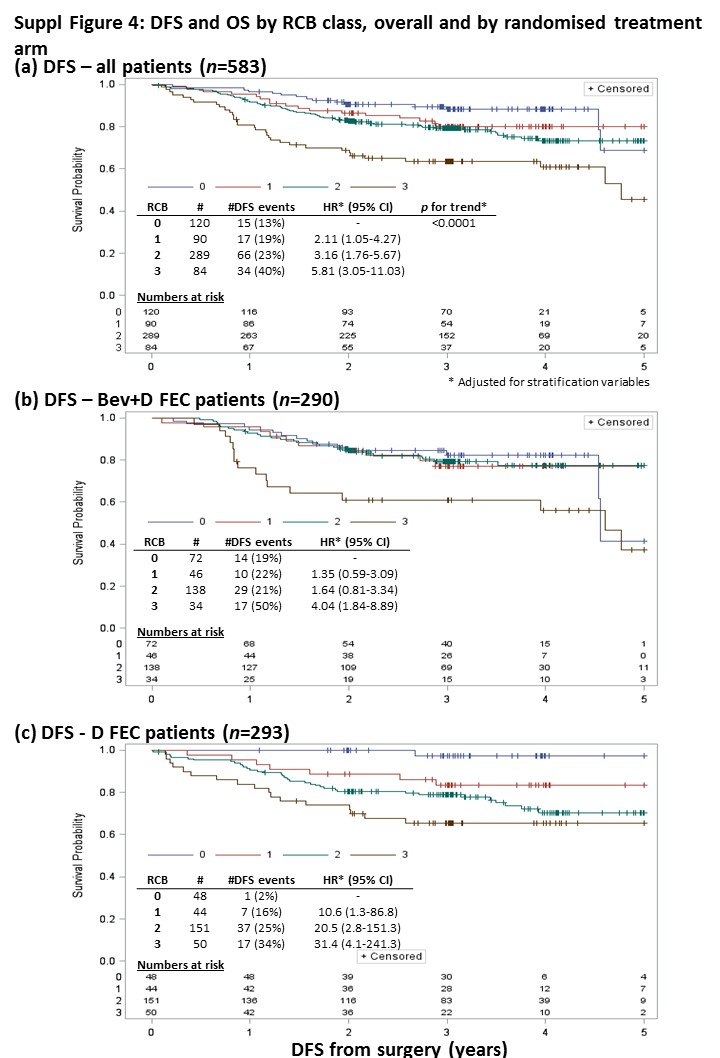

Supplement: Supplementary Figure 4abc [file fig_s4abc_mdx173.jpeg]

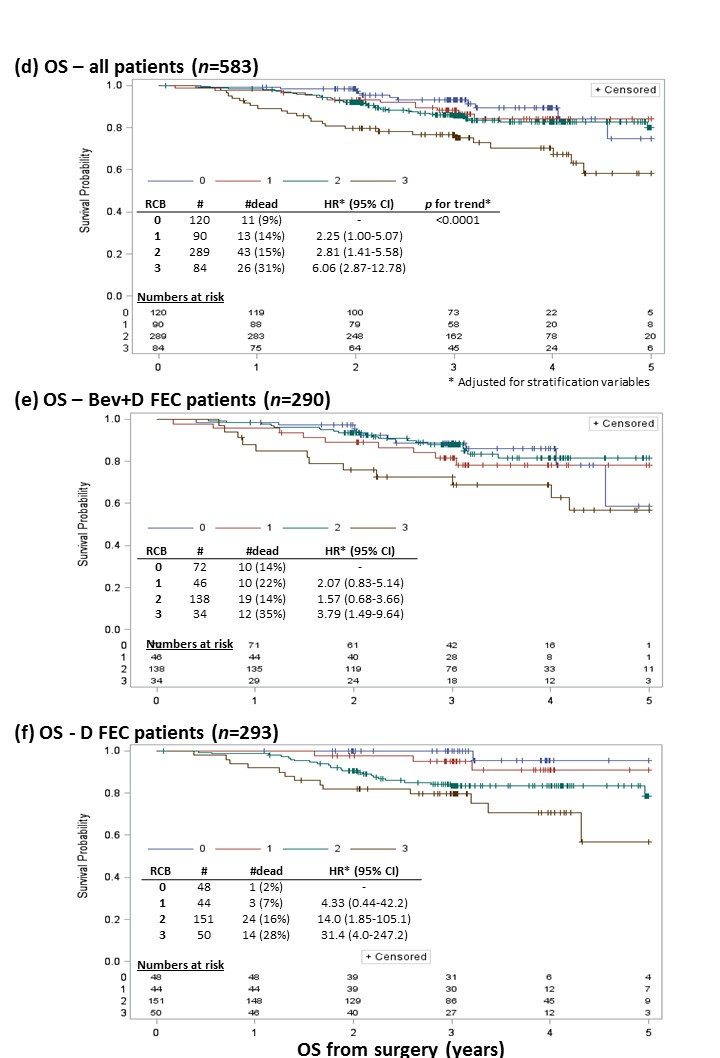

Supplement: Supplementary Figure 4def [file fig_s4def_mdx173.jpeg]

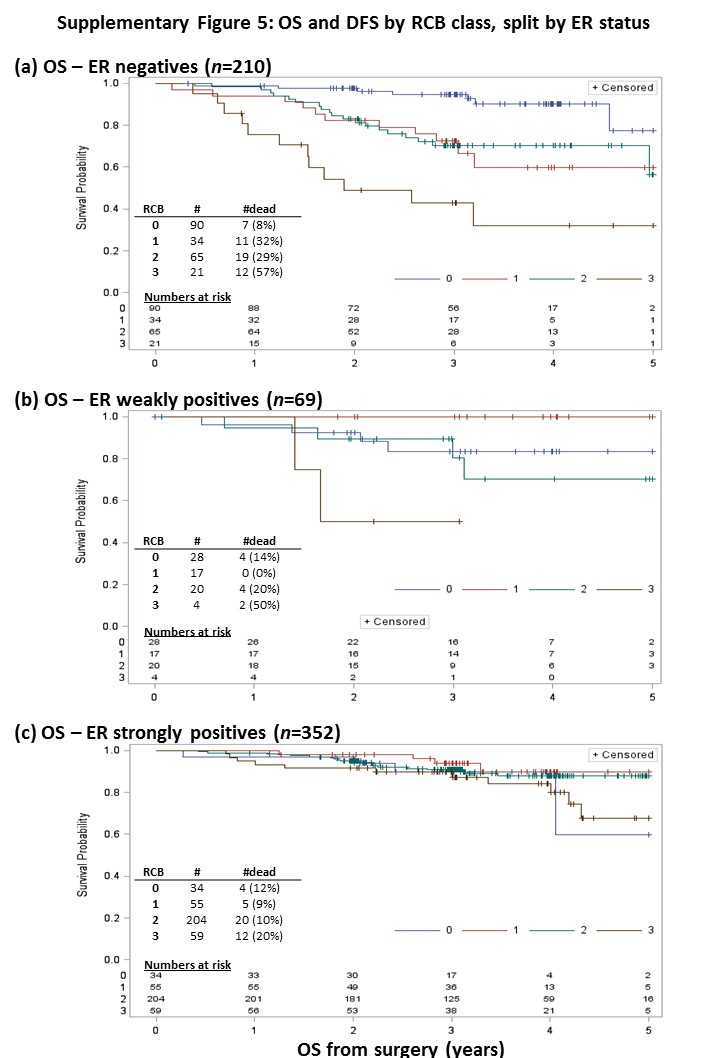

Supplement: Supplementary Figure 5abc [file fig_s5abc_mdx173.jpeg]

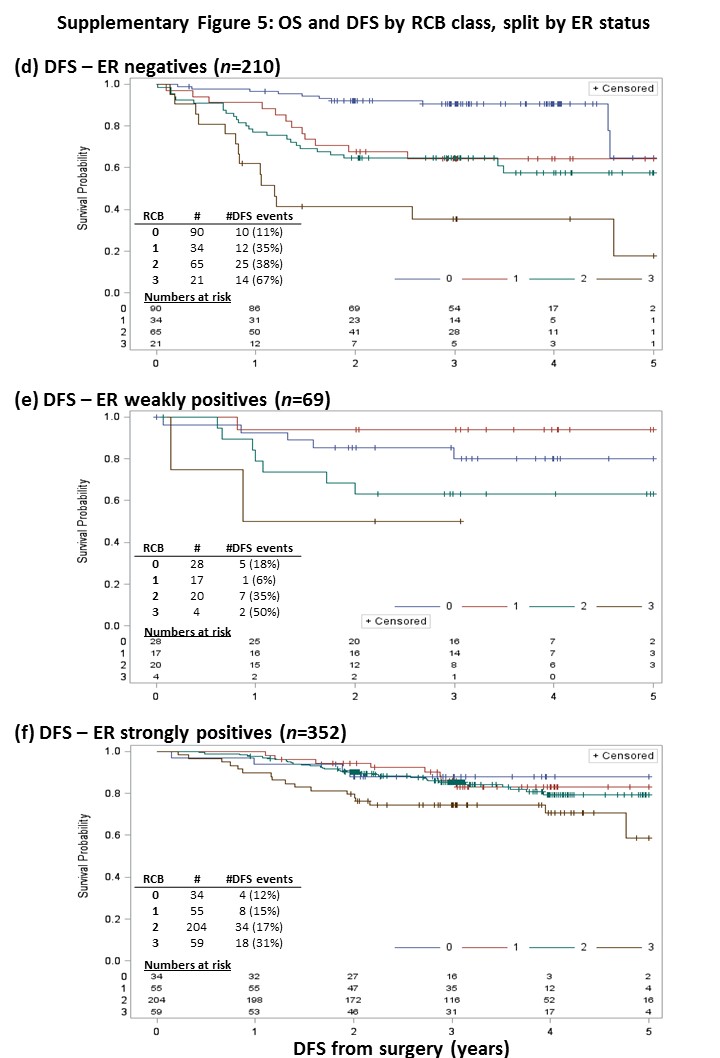

Supplement: Supplementary Figure 5def [file fig_s5def_mdx173.jpeg]
