# Supplementary material for: Disease-free and overall survival at 3.5 years for neoadjuvant bevacizumab added to docetaxel followed by fluorouracil, epirubicin and cyclophosphamide, for women with HER2 negative early breast cancer: ARTemis Trial
Source: Ann Oncol. 2017 Apr 27;28(8):1817–24. doi: 10.1093/annonc/mdx173 (PMC5834079; doi:10.1093/annonc/mdx173)
Supplement: Supplementary Table S1 [file tab_s1.1_mdx173.docx]

**Supplementary Table 1: Patients achieving a pCR, split by DFS event (*n*=129)**

|  | **Bev+D-FEC (*n*=77)** | | **D-FEC (*n*=52)** | |
| --- | --- | --- | --- | --- |
|  | **DFS event** (*n*=16 (21%)) | **No DFS event** (*n*=61 (79%)) | **DFS event** (*n*=3 (6%)) | **No DFS event** (*n*=49 (94%)) |
| **ER status** |  |  |  |  |
| **Neg** | 9 (56%) | 38 (62%) | 2 (67%) | 30 (61%) |
| **Weak pos** | 4 (25%) | 13 (21%) | 1 (33%) | 7 (14%) |
| **Pos** | 3 (19%) | 10 (17%) | - | 12 (25%) |
|  |  |  |  |  |
| **Surgery type *** |  |  |  |  |
| **Mastectomy** | 6 (38%) | 18 (30%) | 1 (33%) | 13 (27%) |
| **Breast Conserving** | 10 (63%) | 43 (70%) | 2 (67%) | 37 (76%) |
| **Re-excision** | - | 1 (2%) | - | 2 (4%) |
| **Reconstruction** | 2 (13%) | 2 (3%) | - | 2 (4%) |
| **Axillary Sampling** | 1 (6%) | 7 (11%) | 1 (33%) | 4 (8%) |
| **Axillary Clearance** | 8 (50%) | 26 (43%) | 2 (67%) | 22 (45%) |
|  |  |  |  |  |
| **Radiotherapy given?** |  |  |  |  |
| **Reported** | 14 (88%) | 52 (85%) | 2 (67%) | 39 (80%) |
| **Not reported** | 2 (12%) | 9 (15%) | 1 (33%) | 10 (20%) |
|  |  |  |  |  |
| **Local** |  |  |  |  |
| **Yes** | 6 (38%) | - | - | - |
| **No** | 10 (62%) | 61 (100%) | 3 (100%) | 49 (100%) |
|  |  |  |  |  |
| **Distant** |  |  |  |  |
| **Yes** | 12 (75%) | - | 3 (100%) | - |
| **No** | 4 (25%) | 61 (100%) | - | 49 (100%) |
|  |  |  |  |  |
| **Dead** |  |  |  |  |
| **Yes** | 12 (75%) | - | 3 (100%) | - |
| **No** | 4 (25%) | 61 (100%) | - | 49 (100%) |
|  |  |  |  |  |
| **Cause of death *** |  |  |  |  |
| **Metastatic Breast Cancer** | 12 (100%) | - | 3 (100%) | - |
| **Acute renal failure** | 1 (8%) | - | - | - |
| **Ovarian second primary** | 1 (8%) | - | - | - |

* Patients can have multiple surgery types and multiple causes of death
